# Teste de Limiar de Desfibrilação e Seguimento a Longo Prazo de Pacientes com Cardiopatia Crônica da Doença de Chagas

**DOI:** 10.36660/abc.20210770

**Published:** 2022-09-30

**Authors:** Marco Paulo Cunha Campos, Luiz Fernando Gouveia Bernardes, João Paulo Chaves de Melo, Lucas Corsino dos Santos, Cristiano Honório Ribeiro Teixeira, Maria Licia Ribeiro Cury Pavão, Elerson Arfelli, Adilson Scorzoni, Anis Rassi, José A. Marin-Neto, André Schmidt

**Affiliations:** 1 Faculdade de Medicina de Ribeirão Preto Universidade de São Paulo Ribeirão Preto SP Brasil Faculdade de Medicina de Ribeirão Preto da Universidade de São Paulo , Ribeirão Preto , SP – Brasil; 2 Anis Rassi Heart Hospital Goiânia GO Brasil Anis Rassi Heart Hospital , Goiânia , GO – Brasil

**Keywords:** Doença de Chagas, Cardiomiopatia Chagásica, Taquicardia Ventricular, Desfibriladores Implantáveis, Cardioversão Elétrica

## Abstract

**Fundamento:**

A morte súbita cardíaca (MSC) é a causa mais comum de óbito na cardiomiopatia crônica da doença de Chagas (CCDC). Visto que muitos pacientes com CCDC que são candidatos a receber um cardioversor desfibrilador implantável (CDI) atendem a critérios que sugerem alto risco de apresentarem limiares de desfibrilação elevados, sugere-se realizar um teste de limite de desfibrilação (LDF).

**Objetivos:**

Investigamos o uso do teste de LDF em pacientes com CCDC, com enfoque nos óbitos relacionados ao implante do CDI e na ocorrência de eventos arrítmicos e o tratamento oferecido durante o seguimento de longo prazo.

**Métodos:**

Avaliações retrospectivas de 133 pacientes com CCDC que receberam CDI, principalmente para prevenção secundária. Foram coletados dados demográficos, clínicos e laboratoriais, escore de Rassi e dados do teste de LDF. Adotou-se p<0,05 como estatisticamente significativo.

**Resultados:**

A média de idade foi 61±13 anos, e 72% da amostra era do sexo masculino. A fração de ejeção basal do ventrículo esquerdo foi 40±15%, e o escore de Rassi médio foi 10±4 pontos. Não ocorreram óbitos durante o teste de LDF, e não foram documentadas falhas do CDI. Foi identificada relação entre escore de Rassi basal mais elevado e LDFs mais elevados (ANOVA =0,007). O tempo médio até o primeiro choque foi de 474±628 dias, mas a aplicação de choque foi necessária em apenas 28 (35%) pacientes com TV, visto que a maioria dos casos se resolveu espontaneamente ou através da programação de ATP. Após seguimento clínico de 1728±1189 dias, em média, ocorreram 43 óbitos, relacionados principalmente a insuficiência cardíaca progressiva e sepse.

**Conclusões:**

Um teste de LDF de rotina pode não ser necessário para pacientes com CCDCs que receberam CDI para prevenção secundária. LDFs elevados parecem ser incomuns e podem estar relacionados a escore de Rassi elevado.

## Introdução

A morte súbita cardíaca (MSC) por taquicardia ventricular (TV) ou fibrilação ventricular (FV) é a causa mais comum de óbito em pacientes com cardiomiopatia crônica da doença de Chagas (CCDC). ^1^ O cardioversor desfibrilador implantável (CDI) tem sido amplamente utilizado e validado em cardiomiopatias isquêmicas e dilatadas, tanto para prevenção primária quanto secundária. O uso empírico do CDI é preconizado para prevenção secundária na CCDC após a recuperação de um evento de MSC ou de TV instável. Uma diretriz definitiva ainda está em discussão. ^2 , 3^ Visto que não existem ensaios clínicos randomizados especificamente sobre o uso de CDI na CCDC, embora alguns estejam em andamento, ^4^ extrapolam-se as diretrizes clínicas para cardiopatia dilatada, apesar de os pacientes com CCDC geralmente apresentarem peculiaridades que levam a apresentações clínicas e patológicas mais graves. A ocorrência de MSC em pessoas jovens e assintomáticas ^5^ é conhecida desde as observações originais de Carlos Chagas sobre a doença que leva seu nome. Além disso, choques inadequados, tempestades elétricas e outras complicações relacionadas ao dispositivo parecem ser mais prevalentes em pacientes com CCDC, pois eles costumam ser mais jovens, têm um estilo de vida mais ativo e são mais propensos a eventos arrítmicos. ^2^ Portanto, a CCDC representa um desafio único quanto à decisão de realizar ou não um teste de limiar de desfibrilação (LDF) antes de implantar o CDI. Nenhuma avaliação sistemática do LDF foi relatada para essa entidade, e a maioria dos estudos nem mesmo comenta se essa avaliação foi realizada ou não. ^6 , 7^ Visto que a maioria dos pacientes com CCDC que são candidatos a receber um CDI apresentam manifestações arrítmicas graves, menor fração de ejeção do ventrículo esquerdo e/ou direito e extensa substituição fibrótica do miocárdio operante, de acordo com a maioria dos escores existentes, os pacientes com CCDC seriam considerados como de alto risco de apresentar LDFs elevados e portanto necessitariam de um teste de LDF. ^8^ Entretanto, em países de baixa renda, onde a necessidade de anestesia geral acarretaria maiores custos e procedimentos mais longos, poderia ser vantajoso evitar a necessidade de avaliação do LDF.

Com os modelos de CDI mais antigos, devido à falta de dados sobre falhas de choque, o teste de LDF era considerado essencial. Esse procedimento não era totalmente previsível, devido às formas de onda monofásicas e ao design e ao posicionamento dos eletrodos, com alguns óbitos e falhas de choque diretamente relacionados ao teste de LDF. ^9^ Aprimoramentos no design dos eletrodos e nas formas de onda de choque levaram a testes de LDF mais seguros. Ao longo dos anos, também houve preocupações relacionadas ao impacto dos choques aplicados durante o teste de LDF na aceleração da disfunção ventricular e no aumento de hospitalizações, então, até recentemente, havia acalorados debates sobre a necessidade de realizar testes de LDF. ^10 , 11^

Dois ensaios clínicos publicados recentemente prestaram esclarecimentos sobre o uso disseminado do teste de LDF em outras doenças que não a CCDC. O estudo SIMPLE ( *Shockless IMPLant Evaluation* ) foi um ensaio clínico randomizado multicêntrico que objetivou avaliar a eficácia e a segurança do teste de LDF no momento do implante do CDI. ^12^ Incluindo aproximadamente 2500 pacientes, o estudo concluiu que o teste não afeta a mortalidade nem prevê falhas de choque. O estudo NORDIC ICD ( *NO Regular Defibrillation testing In Cardioverter defibrillator implantation* ), com delineamento semelhante, avaliou 1077 pacientes e chegou às mesmas conclusões. ^13^

Um estudo que avaliou o teste de LDF em CCDC demonstrou uma alta prevalência de limiares elevados. ^14^ Entretanto, na prática clínica, esses pacientes também responderão bem à terapia de estimulação antitaquicardia ( *anti-tachycardia pacing* , ATP), com redução da necessidade da aplicação de choques. ^15^ Dessa forma, investigamos o uso do teste de LDF em pacientes com CCDC, com foco nos óbitos relacionados ao implante do CDI e na ocorrência de eventos arrítmicos e no tratamento oferecido durante o seguimento de longo prazo.

## Métodos

Realizamos uma avaliação retrospectiva de pacientes com CCDC que receberam um CDI no Hospital das Clínicas da Faculdade de Medicina de Ribeirão Preto-USP, Brasil, entre 2001 e 2019. Todos os pacientes tiveram dois testes sorológicos positivos para doença de Chagas. Foram coletados dados demográficos (idade, gênero), clínicos (indicação de CDI, classe funcional da *New York Heart Association* , dados ecocardiográficos, características do ECG, medicamentos em uso no momento do implante do CDI) e escore de Rassi. ^16^ O estudo foi aprovado pelo Comitê de Ética da nossa instituição (CAAE:52530116.8.0000.5440).

Foi realizado um teste de LDF para todos os pacientes conforme o protocolo e os dados foram coletados. Durante os anos da coleta de dados, a rotina do teste de LDF foi alterada, devido ao aumento na experiência da equipe e a novas informações na literatura sobre as consequências do teste. Em geral, a provocação da arritmia ventricular era obtida com um choque cronometrado durante o registro da onda T. Em dispositivos da St. Jude Medical, a indução da arritmia foi obtida com estimulação por corrente contínua. Em caso de falha após duas tentativas de indução de arritmia, foi aplicada estimulação em rajada de 50 Hertz. Por fim, em caso de falha após mais duas tentativas, o teste de LDF foi finalizado e o dispositivo programado para fornecer a quantidade máxima de energia.

Nos primeiros anos do estudo, o primeiro choque de desfibrilação interna era programado para fornecer 15 Joules, seguido de uma tentativa com 20 Joules. Se a desfibrilação fosse malsucedida, era aplicada desfibrilação interna com energia máxima e, se a arritmia persistisse após duas tentativas, era aplicado choque externo e, depois disso, o eletrodo era reposicionado. Nesse protocolo inicial, se a primeira tentativa com 15 Joules fosse bem-sucedida, era testado um choque de 10 Joules.

Ao longo dos anos, a energia máxima fornecida pelos CDIs aumentou, e foi definida uma margem de segurança de 10 Joules para o primeiro choque bem-sucedido durante o teste de LDF. Além disso, estabeleceu-se que o primeiro choque do teste de LDF fosse programado para 20 Joules e, se bem-sucedido, o teste de LDF seria concluído. Se fosse malsucedido, tentava-se uma carga de 25 Joules, seguida do reposicionamento do eletrodo. Um LDF elevado foi definido como < 10J da margem de segurança.

Os parâmetros do CDI foram coletados no momento do implante, os pacientes foram acompanhados a cada três a seis meses, e foram registrados a duração e o tipo dos eventos arrítmicos, assim como a terapia oferecida e a sua eficácia.

### Análise estatística

As variáveis contínuas foram expressas como média ± desvio padrão, caso apresentassem distribuição normal. A normalidade dos dados foi examinada por histogramas e pelo teste de Shapiro-Wilk. As variáveis qualitativas foram expressas como valores absolutos e percentuais e comparadas utilizando o teste qui-quadrado de tendência ou o teste exato de Fisher. Foi utilizada análise de variância de um fator ( *one-way* ANOVA), seguida pelo pós-teste de Bonferroni, para comparar a relação entre o escore de Rassi e os valores do teste de LDF. Utilizamos o pacote estatístico SPSS, versão 25 (IBM Corp., Armonk, EUA), e a significância estatística foi definida como p < 0,05.

## Resultados

Foram incluídos 133 pacientes com CCDC que receberam um CDI. A média de idade foi 61±13 anos, e 72% da amostra era do sexo masculino. A média da fração de ejeção do ventrículo esquerdo (FEVE) foi 40±15%, e o diâmetro diastólico médio do ventrículo esquerdo foi de 61±10 mm antes do implante. A escore de Rassi médio foi de 10±4 pontos. A grande maioria (120 pacientes – 90,2%) recebeu o dispositivo para prevenção secundária. TV documentada foi o motivo para o implante do CDI em aproximadamente metade da amostra, seguida de MSC abortada. A Tabela 1 apresenta as principais indicações clínicas para CDI em nossa amostra e um resumo dos dados demográficos, clínicos e laboratoriais. A Tabela 2 resume os dados demográficos e laboratoriais distribuídos de acordo com os tercis do escore de Rassi, demonstrando tendência para choques mais precoces à medida que o tercil do escore de Rassi aumenta.

**Tabela 1:** Dados basais demográficos, clínicos e laboratoriais antes do implante do CDI nos 133 pacientes com cardiomiopatia crônica de doença de Chagas incluídos

Idade (anos)	61±13
Sexo masculino – N(%)	96 (72,2)
Rassi score	10,2±4,2
Hipertensão sistêmica – N(%)	46(34,6)
Diabetes melito – N(%)	11(8,3)
Insuficiência renal crônica – N(%)	22(16,5)
**Classe funcional da NYHA – N(%)**	
I	48(36,1)
II	52(39,1)
III	28(21,1)
IV	03(2,3)
N/D	02(1,5)
**Medicamentos – N(%)**	
IECA	84(63,2)
Betabloqueadores	100(75,2)
Diuréticos	75(56,4)
BRA	23(17,3)
Amiodarona	93(69,9)
Anticoagulante oral	33(24,8)
**Ritmo no eletrocardiograma – N(%)**	
Sinusal	104(78,2)
Fibrilação atrial	8(6,0)
Marcapasso	21(15,8)
**Dados ecocardiográficos**	
FEVE(%)	40±15
Diâmetro diastólico final do ventrículo esquerdo (mm)	61±10
Dimensão do átrio esquerdo (mm)	47±9
**Indicação para CDI – N(%)**	
Primário	13 (9,8)
Taquicardia ventricular documentada	66 (49,6)
Morte súbita cardíaca abortada	28 (21,1)
Síncope	21 (15,8)
Fibrilação ventricular documentada	2 (1,5)
Quase síncope	2 (1,5)
Palpitações	1 (0,8)

*NYHA: New York Heart Association; N/D: não disponível; IECA: Inibidores da enzima conversora de angiotensina; BRA: Bloqueadores dos receptores de angiotensina; FEVE: Fração de ejeção do ventrículo esquerdo.*

**Tabela 2:** Distribuição dos parâmetros demográficos, laboratoriais e de seguimento de acordo com o tercil do escore de Rassi

Variável	Tercil 1 do escore de Rassi (n=32)	Tercil 2 do escore de Rassi (n=53)	Tercil 3 do escore de Rassi (n=46)	Anova valor de p
Idade (anos)	60 ± 11	62 ± 12	60 ± 14	0,788
Sexo masculino (%)	75	62	83	0,062 *
Escore de Rassi	4,97 ± 1,26	9,20 ± 1,19	14,93 ± 2,27	<0,001
FEVE (%)	44 ± 11	40 ± 15	36 ± 16	0,065
DDVE (mm)	57 ± 7	60 ± 10	65 ± 11	0,002
Teste de choque (J)	18,2 ± 3,1	18,8 ± 2,6	20,5 ± 4,2	0,007
Tempo até o primeiro choque (dias)	807 ± 964	410 ± 609	395 ± 412	0,071

*: teste qui-quadrado de tendência. FEVE: fração de ejeção do ventrículo esquerdo; DDFVE: diâmetro diastólico final do ventrículo esquerdo.

Não ocorreram óbitos durante o procedimento de implante e o teste de LDF.

**Figura 1:**
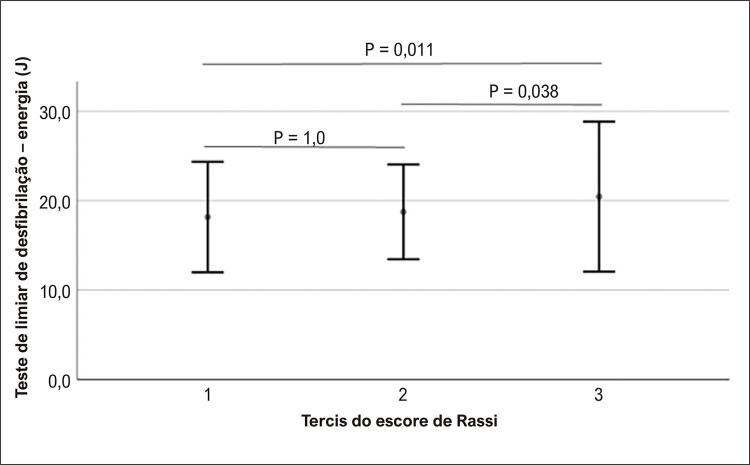
Valores do teste de limiar de desfibrilação expressos como média e desvio padrão de acordo com tercis do escore de Rassi, demonstrando um aumento progressivo nos valores conforme o escore de Rassi aumenta (one-way ANOVA= 0,007) e pós-testes de Bonferroni nos quais a principal diferença ocorre entre o terceiro tercil e os outros dois, que são estatisticamente semelhantes.

**Figura 2:**
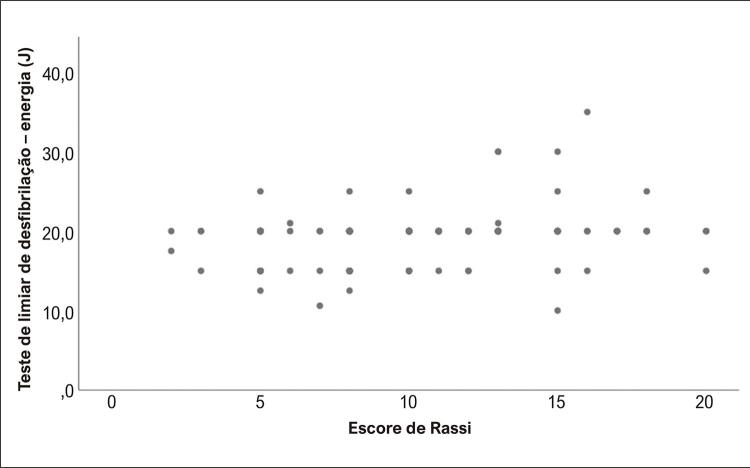
Distribuição dos valores do teste de limite de desfibrilação (LDF) de acordo com o escore de Rassi. É possível observar que os pacientes com LDF elevado apresentaram um escore de Rassi igual ou maior do que 13. Observação: Um ponto pode representar mais do que um paciente.

No seguimento, o tempo médio até o primeiro choque foi de 474±628 dias. Cem pacientes receberam algum tratamento com CDI, 79 para TV e 21 para FV. O primeiro choque, definido em 20J, foi eficaz em 88 pacientes. Um valor menor foi obtido em 25% dos casos, e um valor maior foi necessário em 12 (9%) pacientes. Foram identificados valores elevados no teste de LDF (≥30J) em 4 (3%) pacientes. Alguns pacientes foram programados para receber valores tão baixos como 10J (1 paciente) ou tão elevados quanto 35J (1 paciente). A Figura 1 apresenta a relação entre o escore de Rassi e os valores basais do teste de LDF, sugerindo que um escore mais elevado indicou LDFs mais elevados (ANOVA =0,007). Todos os pacientes classificados com LDF elevado apresentaram escore de Rassi ≥13 pontos ( Figura 2 ).

Foi necessária a aplicação de choque em apenas 2 (35%) pacientes com TV, pois a maioria dos casos se resolveu espontaneamente ou através de ATP programada para ser fornecida antes do choque, e em apenas 4 (14%) eventos de TV foram necessários múltiplos choques. Com relação aos pacientes com FV, apenas 4 (19%) receberam mais de uma descarga. Após um seguimento clínico de 1728±1189 dias, em média, ocorreram 43 óbitos, relacionados principalmente a insuficiência cardíaca progressiva e sepse. Nenhum óbito pôde ser atribuído a falhas do CDI.

## Discussão

Nosso estudo apresenta dados sobre o uso sistemático do teste de LDF especificamente em pacientes com cardiomiopatia dilatada comumente associada a MSC, a qual ocorre principalmente, mas não exclusivamente, no contexto clínico de FEVE baixa devido ao comprometimento fibrótico generalizado do coração. Vários marcadores de LDF elevado estiveram presentes, mas cabe destacar que aproximadamente metade da população da amostra foi composta de pessoas abaixo dos 60 anos e que a maioria era do sexo masculino e apresentava FEVE baixa (<40%). Portanto, nossa amostra pode ser definida como propensa a complicações hospitalares e LDF elevadosao serem aplicados escores existentes. ^8 , 17^ Também é relevante apontar que prevenção secundária foi o principal motivo para implante do CDI, pois ainda não foram estabelecidas diretrizes para prevenção primária na CCDC, e são necessários estudos randomizados prospectivos em pacientes com escores de Rassi elevados.

Nosso protocolo para o teste de LDF evoluiu ao longo de duas décadas, refletindo os avanços na tecnologia dos CDIs, conforme relatado anteriormente. ^18^ Identificamos que apenas 3% da nossa amostra apresentava LDF elevado, resultado próximo ao dos valores mais baixos encontrados na literatura, entre 2,2 e 12%. ^10^ Esse achado original indica que, embora apresentem miocárdio com fibrose extensa, os pacientes com CCDC podem não representar uma população que necessite de muitos ajustes durante um procedimento de implante de CDI.

Outra informação relevante obtida a partir da nossa amostra é que nenhum óbito foi relacionado ao procedimento em pacientes com CCDC, corroborando os resultados de registros prévios de outro países relatando baixa incidência de complicações relacionadas ao procedimento, ^9^ e de uma revisão sistemática recente, a qual declarou que “não há relatos consistentes de óbitos relacionados ao implante.” ^19^

Nossos resultados também demonstram que TV foi a arritmia potencialmente letal predominante em pacientes com CCDC e que a ATP foi capaz de restaurar o ritmo cardíaco na maioria dos casos, uma característica que está de acordo com relatos anteriores na população com CCDC. ^6 , 20^ Isso reforça a ideia de que um protocolo de ATP bem estabelecido é essencial para possibilitar a restauração do ritmo cardíaco sem choques evitáveis, especialmente quando se considera que a maioria dos pacientes com CCDC apresentam alta prevalência de episódio de tempestade elétrica. ^21^

Nosso resultado original que identificou uma relação significativa entre o escore de Rassi para mortalidade global e LDF pode sugerir que os pacientes com escore de Rassi elevado são os que realmente precisam realizar o teste de LDF, mas uma definição clara desse ponto requer mais evidências provenientes de um ensaio clínico maior.

É tranquilizador relatar que o acompanhamento do nosso estudo demonstrou que não foram observadas falhas no dispositivo, em conformidade com relatos prévios independentes. Por fim, visto que os pacientes com CCDC que sobrevivem ao risco ameaçador da MSC frequentemente evoluem para insuficiência cardíaca progressiva ou óbito devido a outras complicações clínicas, parece razoável presumir que, mesmo com o aumento da fibrose e da disfunção do VE, os CDIs podem preservar a sua capacidade de prevenir MSC.

Nosso estudo apresenta algumas limitações. Em primeiro lugar, embora seja uma das maiores disponíveis, a nossa amostra é proveniente de um único centro. Além disso, as alterações no protocolo do teste de LDF de acordo com as inerentes melhorias técnicas e avanços no conhecimento certamente influenciaram nossos resultados, mas isso não pode ser controlado, devido a questões éticas. Por fim, não podemos traduzir nossos resultados para pacientes com CCDC que podem receber um CDI para prevenção primária.

## Conclusões

Nossos dados indicam que o teste de LDF de rotina pode não ser necessário para pacientes com cardiomiopatia de doença de Chagas que receberam CDI para prevenção secundária. LDFs elevados parecem ser incomuns e podem estar relacionados a um escore de Rassi elevado. TV responsiva a ATP é a forma mais comum de AV, e a maioria dos eventos de FV são tratados adequadamente com um choque. Além disso, considerando as limitações de recursos em países onde a CCDC é endêmica, é provavelmente mais custo-efetivo não realizar o teste de LDF.
