# Supplementary material for: Teste de Limiar de Desfibrilação e Seguimento a Longo Prazo de Pacientes com Cardiopatia Crônica da Doença de Chagas
Source: Arq Bras Cardiol. 2022 Sep 30;119(6):923–8. [Article in Portuguese] doi: 10.36660/abc.20210770 (PMC9814814; doi:10.36660/abc.20210770)
Supplement: Translation

# Translation: Defibrillation Threshold Testing and Long-term Follow-up in Chagas Disease

**Authors:** Marco Paulo Cunha Campos, Luiz Fernando Gouveia Bernardes, João Paulo Chaves de Melo, Lucas Corsino dos Santos, Cristiano Honório Ribeiro Teixeira, Maria Licia Ribeiro Cury Pavão, Elerson Arfelli, Adilson Scorzoni, Anis Rassi, José A. Marin-Neto, André Schmidt

**Affiliations:** 1 Brazil Faculdade de Medicina de Ribeirão Preto da Universidade de São Paulo, Ribeirão Preto, SP – Brazil; 2 Brazil Anis Rassi Heart Hospital, Goiânia, GO – Brazil

**Keywords:** Chagas Disease, Chagas Cardiomyopathy, Tachycardia, Ventricular, Defibrillators, Implantable, Electric Countershock

## Abstract

**Background:**

Sudden cardiac death is the most common cause of death in chronic Chagas cardiomyopathy (CCC). Because most CCC patients who are candidates for implantable cardioverter-defibrillators (ICD) meet criteria for high defibrillation threshold values, a defibrillator threshold test (DTT) is suggested.

**Objectives:**

We investigated the use of DTT in CCC patients, focusing on deaths related to ICD and arrhythmic events, as well as treatment during long-term follow-up.

**Methods:**

We retrospectively evaluated 133 CCC patients who received an ICD mainly for secondary prevention. Demographic, clinical, laboratory data, Rassi score, and DTT data were collected, with p < 0.05 considered significant.

**Results:**

The mean patient age was 61 (SD, 13) years and 72% were men. The baseline left ventricular ejection fraction was 40 (SD, 15%) and the mean Rassi score was 10 (SD, 4). No deaths occurred during DTT and no ICD failures were documented. There was a relationship between higher baseline Rassi scores and higher DTT scores (ANOVA = 0.007). The mean time to first shock was 474 (SD, 628) days, although shock was only necessary for 28 (35%) patients with ventricular tachycardia, since most cases resolved spontaneously or through antitachycardia pacing. After a mean clinical follow-up of 1728 (SD, 1189) days, 43 deaths occurred, mainly related to progressive heart failure and sepsis.

**Conclusions:**

A routine DTT may not be necessary for CCC patients who receive an ICD for secondary prevention. High DTT values seem to be unusual and may be related to high Rassi scores.

## Introduction

Sudden cardiac death (SCD) due to ventricular tachycardia (VT) or ventricular fibrillation is the most common cause of death in patients with chronic Chagas cardiomyopathy (CCC).^1^

Implantable cardioverter-defibrillators (ICD) have been extensively used and validated in ischemic and dilated cardiomyopathies, both for primary and secondary prevention. ICDs are recommended for secondary prevention in CCC after recovery from SCD or an unstable VT event. Definitive guidelines have not yet been produced^2 , 3^ since no specific randomized trials for ICD in CCC are available, although some are ongoing.^4^ Clinical guidelines for dilated cardiomyopathies have been inferred, although CCC usually involves peculiarities that lead to more severe clinical and pathological presentations. The occurrence of SCD in young and asymptomatic patients^5^ has been known since Carlos Chagas’ original observations of the disease. In addition, inappropriate shocks, electrical storms, and other device-related complications seem more prevalent in CCC patients, since they are usually younger, have a more active lifestyle, and have a higher propensity to arrhythmic events.^2^ Hence, CCC presents a unique challenge regarding defibrillation threshold testing (DTT) before ICD implantation. No systematic evaluation of DTT has been published and most studies do not report whether it was performed or not.^6 , 7^ Because most CCC patients who are ICD candidates have severe arrhythmic manifestations, low left and/or right ventricular ejection fraction, and extensive fibrotic replacement of working myocardium, CCC patients would be considered at risk of high defibrillation threshold values and would thus require DTT.^8^ However, in low-income countries, where the need for general anesthesia would imply higher costs and longer procedures, avoiding DTT could be advantageous.

Due to a lack of data on shock failures, DTT was considered essential for earlier ICD models. This procedure was not completely predictable due to monophasic waveforms and lead design and placement, with some deaths and shock failures directly attributable to the test.^9^ Refinements in lead design and shock waveforms led to safer DTT. Over the years, there have also been concerns that the shocks delivered during DTT accelerate ventricular dysfunction and cause more hospitalizations, so until recently, there was controversy about the need to perform DTTs.^10 , 11^

Two recently published trials clarified the appropriateness of DTT for diseases other than CCC. SIMPLE (Shockless IMPLant Evaluation) was a randomized multicenter trial to evaluate the efficacy and safety of DTT when implanting an ICD.^12^ With a sample of nearly 2500 patients, the trial concluded that DTT neither would affect mortality nor predict shock failures. The NORDIC ICD (NO Regular Defibrillation testing In Cardioverter defibrillator implantation) trial, with a similar design, evaluated 1077 patients and reached the same conclusions.^13^

A study that evaluated DTT in CCC showed a high prevalence of high threshold values.^14^ However, in clinical practice, those patients also respond well to antitachycardia pacing, having less need for shocks.^15^ Hence, we investigated the use of DTT in CCC patients, focusing on ICD-related deaths and arrhythmic events and treatment during long-term follow-up.

## Methods

We conducted a retrospective evaluation of CCC patients who received an ICD at the Hospital das Clínicas da Faculdade de Medicina da Universidade de São Paulo–Ribeirão Preto, Brazil, between 2001 and 2019. All patients had two positive serological tests for Chagas disease. Demographic (age, sex), clinical (ICD indication, functional class, echocardiographic data, electrocardiogram characteristics, medications in use at ICD implantation), and Rassi score^16^ data were collected. The study was approved by our institution’s ethics committee (CAAE:52530116.8.0000.5440).

DTT was performed for all patients per protocol and the data were collected. During the data collection period, the DTT routine changed due to greater team experience and new literature about its consequences. In general, ventricular arrhythmia was obtained with a timed shock during T-wave recording. Using St. Jude Medical devices, arrhythmia was induced with a continuous current. If arrhythmia could not be induced after two attempts, 50-Hertz burst pacing was applied. Finally, if 2 further attempts failed, DTT was finalized and the device was programmed for maximum energy.

In the initial years of the study, the first internal defibrillation shock was programmed for 15 Joules, followed by 20 Joules. If defibrillation was unsuccessful, internal defibrillation at maximum energy was attempted, and if the arrhythmia persisted after 2 attempts, an external shock was delivered and the electrode was repositioned. In this initial protocol, if the 15-Joule attempt was successful, a 10-Joule shock was attempted.

Over the years, the maximum energy delivered by ICDs was increased and a 10-Joule safety margin was determined for the first successful shock during DTT. It was also determined that the first DTT shock was programmed for 20 Joules and, if successful, the DTT test was terminated. If unsuccessful, a 25-Joule shock was attempted, followed by repositioning of the electrode. A high DTT value was defined as < 10 Joules from the safety limit.

ICD parameters were collected at the time of implantation, and patients were followed up every 3 to 6 months regarding the time and type of arrhythmic events, as well as the therapy received and its efficacy.

### Statistical analysis

Continuous variables were expressed as mean (standard deviation) if normally distributed. Data normality was assessed using histograms and the Shapiro-Wilk test. Qualitative variables were expressed as absolute values and percentages and were compared using chi-square for trend or Fisher’s exact test. Analysis of variance (One-way ANOVA) with Bonferroni adjustment was used as a post-test to compare the relationship between Rassi score and DTT values. We used IBM SPSS Statistics version 25 (IBM Corp., Armonk, NY, USA) and a significance level of p < 0.05.

## Results

We included a total of 133 CCC patients who received an ICD. The mean age was 61 (SD, 13) years and 72% were men. The mean left ventricular ejection fraction was 40 (SD, 15%) and the mean left ventricle diastolic diameter was 61 (SD, 10 mm) before implantation. The mean Rassi score was 10 (SD, 4). The vast majority of patients (120, 90.2%) received the device for secondary prevention. Documented VT was the reason for implantation in nearly half of the sample, followed by aborted sudden cardiac death. Table 1 presents the main clinical indications for an ICD in our sample and a summary of the demographic, clinical, and laboratory data. Table 2 summarizes the demographic and laboratory data according to Rassi tertiles, showing a trend toward earlier shocks with higher Rassi tertiles.

**Table 1:** Baseline demographic, clinical, and laboratory data before implantable cardioverter-defibrillator implantation in 133 chronic Chagas cardiomyopathy patients

Age (years)	61±13
Male – N(%)	96 (72.2)
Rassi score	10.2±4.2
Systemic Hypertension –N(%)	46(34.6)
Diabetes Mellitus – N(%)	11(8.3)
Chronic Renal Failure – N(%)	22(16.5)
**NYHA Class – N(%)**	
I	48(36.1)
II	52(39.1)
III	28(21.1)
IV	03(2.3)
N/D	02(1.5)
**Medications – N(%)**	
ACEI	84(63.2)
Beta-blockers	100(75.2)
Diuretics	75(56.4)
ARB	23(17.3)
Amiodarone	93(69.9)
Oral anticoagulants	33(24.8)
**Electrocardiogram Rhythm – N(%)**	
Sinus	104(78.2)
Atrial fibrillation	8(6.0)
Pacemaker	21(15.8)
**Echocardiographic data**	
LVEF(%)	40±15
Left Ventricle End Diastolic Diameter (mm)	61±10
Left Atrium dimension (mm)	47±9
**ICD indication – N(%)**	
Primary	13 (9.8)
Documented Ventricular Tachycardia	66 (49.6)
Aborted sudden cardiac death	28 (21.1)
Syncope	21 (15.8)
Documented Ventricular Fibrillation	2 (1.5)
Near Syncope	2 (1.5)
Palpitations	1 (0.8)

*NYHA: New York Heart Association; N/A: not available; ACEI: Angiotensin-Converting Enzyme Inhibitors; ARB: Angiotensin Receptor Blockers; ICD: implantable cardioverter-defibrillator; LVEF: Left ventricle ejection fraction.*

**Table 2:** Distribution of demographic, laboratory, and follow-up parameters according to Rassi tertile

Variable	Rassi tertile 1 (n=32)	Rassi tertile 2 (n=53)	Rassi tertile 3 (n=46)	Anova p-value
Age (years)	60 ± 11	62 ± 12	60 ± 14	0.788
Male (%)	75	62	83	0.062 *
Rassi score	4.97 ± 1.26	9.20 ± 1.19	14.93 ± 2.27	<0.001
LVEF (%)	44 ± 11	40 ± 15	36 ± 16	0.065
LVDD (mm)	57 ± 7	60 ± 10	65 ± 11	0.002
Shock Test (J)	18.2 ± 3.1	18.8 ± 2.6	20.5 ± 4.2	0.007
Time to first shock (days)	807 ± 964	410 ± 609	395 ± 412	0.071

* = chi-square test for trend. LVEF: left ventricular ejection fraction; LVDD: left ventricular end diastolic dimension.

No deaths occurred during the implantation procedure or DTT.

At follow-up, the mean time to the first shock was 474 (SD, 628) days. A total of 100 patients received some ICD treatment (79 for VT and 21 for ventricular fibrillation). The first shock, set at 20 Joules, was effective in 88 patients. A lower value was obtained in 25% of the cases and a higher value was necessary for 12 (9%) patients. High DTT values (≥ 30 Joules) were identified in 4 (3%) patients. Programming ranged from 10 (1 patient) to 35 Joules (1 patient). Figure 1 depicts the relationship between Rassi score and baseline DTT scores, showing the association between higher Rassi scores and a higher DTT scores (ANOVA = 0.007). All patients with high DTT scores had a Rassi score ≥ 13 points ( Figure 2 ).

**Figure 1:** Defibrillation threshold test scores expressed as mean and standard deviation according to Rassi score tertiles, showing a progressive increase with higher Rassi scores (one-way ANOVA= 0.007) and Bonferroni post-tests, in which the main difference was between the third tertile, with the other two being statistically similar.

**Figure 2:** Defibrillation threshold test score distribution according to Rassi score. As can be seen, patients with high scores had a Rassi score ≥ 13. Note: Each point may represent more than one patient.

Only 2 (35%) VT patients required a shock, with most cases resolving spontaneously or through antitachycardia pacing programmed before the shock; multiple shocks were required in only 4 (14%) VT events. Only 4 (19%) ventricular fibrillation patients received more than 1 shock. After a mean clinical follow-up of 1728 (SD, 1189) days, 43 deaths occurred, which were mainly related to progressive heart failure and sepsis. No deaths could be attributed to ICD failure.

## Discussion

Our study presents data on the systematic use of DTT in patients with dilated cardiomyopathy, which is commonly associated with SCD and occurs mostly (but not exclusively) in a clinical setting of low left ventricular ejection fraction due to widespread fibrotic remodeling of the heart. Various high DTT score markers were present, but it should be pointed out that nearly half of the sample was < 60 years of age most were men with low left ventricular ejection fraction (< 40%). Hence, our population may be defined as at risk of in-hospital complications and high DTT scores.^8 , 17^ It is also relevant that secondary prevention was the main reason for ICD implantation, since no guidelines have yet been established for primary prevention in CCC, and prospective randomized studies are needed in patients with high Rassi scores.

Our DTT protocol has evolved over two decades, reflecting the advancing technology of ICDs, as previously reported.^18^ Only 3% of our sample had high DTT scores, close to the lowest values found in the literature (2.2 to 12%).^10^ This original finding indicates that although having an extensively fibrotic myocardium, CCC patients may not demand many adjustments during an ICD procedure.

Another relevant finding in our cohort is that no deaths could be attributed to the procedure, corroborating the low incidence of procedure-related complications found in different countries,^9^ as well as the results of a recent systematic review that “implant-related deaths are not consistently reported.^19^

Our results also demonstrate that VT was the most predominant potentially lethal arrhythmia in CCC patients and that antitachycardia pacing successfully restored rhythm in most cases, which agrees with previous reports in populations with CCC.^6 , 20^ This reinforces the view that a well-established antitachycardia pacing protocol is essential for restoring rhythm without unnecessary shocks, especially since most CCC patients have a high prevalence of electrical storms.^21^

We identified a significant relationship between Rassi scores for overall mortality and DTT scores, which suggests that patients with high Rassi scores may actually need DTT; however, a larger trial is required to clarify this point.

It is reassuring that our follow-up showed no device failures, which is in line with previous independent reports. Finally, since the clinical course of CCC patients who survive SCD frequently involves progressive severe heart failure or death due to other clinical complications, it seems reasonable to assume that even with increased LV fibrosis and dysfunction, ICDs may continue to prevent SCD.

Our study has some limitations. First, although it is one of the largest available, our sample is from a single center. In addition, our DTT protocol changed due to improved knowledge and technical advances, which certainly influenced our results but could not be controlled due to ethical concerns. Finally, our results cannot be translated to patients with CCC who receive an ICD for primary prevention.

## Conclusions

Our data indicate that routine DTT may not be necessary for chronic Chagas cardiomyopathy patients who receive an ICD for secondary prevention. High DTT scores seem to be unusual and may be related to high Rassi scores. VT responsive to antitachycardia pacing is the most common form of ventricular arrhythmia and most ventricular fibrillation events are adequately treated with 1 shock. In addition, considering the limited resource in countries where CCC is endemic, it is probably cost-effective to skip DTT.
